# Matrix sketching framework for linear mixed models in association studies

**DOI:** 10.1101/gr.279230.124

**Published:** 2024-09

**Authors:** Myson Burch, Aritra Bose, Gregory Dexter, Laxmi Parida, Petros Drineas

**Affiliations:** 1 Computational Genomics, IBM T.J. Watson Research Center, Yorktown Heights, New York 10598, USA;; 2 Computer Science Department, Purdue University, West Lafayette, Indiana 47907, USA

## Abstract

Linear mixed models (LMMs) have been widely used in genome-wide association studies to control for population stratification and cryptic relatedness. However, estimating LMM parameters is computationally expensive, necessitating large-scale matrix operations to build the genetic relationship matrix (GRM). Over the past 25 years, Randomized Linear Algebra has provided alternative approaches to such matrix operations by leveraging *matrix sketching*, which often results in provably accurate fast and efficient approximations. We leverage matrix sketching to develop a fast and efficient LMM method called Matrix-Sketching LMM (MaSk-LMM) by sketching the genotype matrix to reduce its dimensions and speed up computations. Our framework comes with both theoretical guarantees and a strong empirical performance compared to the current state-of-the-art for simulated traits and complex diseases.

Linear mixed models (LMMs) are widely used when conducting genome-wide association studies (GWAS) for complex traits in the presence of population structure. It is well known that population structure plays an important role in confounding results and generating false positive associations (Yang et al. 2010). LMMs are able to capture and correct such confounders in the data, while decomposing phenotypic correlations into genetic and nongenetic components. These desirable properties have resulted in wide use of LMMs in GWAS and genomic selection problems in human and plant genetics, as well as in other biological applications (Lippert et al. 2011; Yang et al. 2011; Runcie and Crawford 2019; Runcie et al. 2021; Yamamoto and Matsunaga 2021).

On the negative side, LMMs have well-known limitations that we attempt to address in our work. Most prominent among those limitations are the increased computational requirements in terms of computational time and memory space that these models necessitate. Computing LMM parameters involves building a genetic relationship matrix (GRM) to account for genome-wide sample structure; estimating the phenotypic variance using a random-effects model; and computing association statistics that account for the variance. Let *m* be the number of single-nucleotide polymorphisms (SNPs) or genetic markers and let *n* be the number of individuals. Then, LMMs require multiple $O(n^{3})$ or $O(mn^{2})$ matrix operations such as large matrix inversions, multiplications, etc. Such operations make straightforward LMM computations intractable for large biobanks and create a need for methods that reduce the computational cost of LMM association analyses. Several methods have been developed to achieve computational speedups: Prominent among those are EMMAX (Kang et al. 2010), FaST-LMM (Lippert et al. 2011), GEMMA (Zhou and Stephens 2012), GRAMMAR-Gamma (Svishcheva et al. 2012), GCTA (Yang et al. 2011), BOLT-LMM (Loh et al. 2015), Regenie (Mbatchou et al. 2021), fastGWA (Jiang et al. 2019), and SAIGE (Zhou et al. 2020). Some of these methods estimate the LMM variance parameter exactly and obtain speedups using spectral decompositions of the GRM (Kang et al. 2010) via block optimizations (Lippert et al. 2011). Other methods perform approximate variance estimation (Kang et al. 2010; Svishcheva et al. 2012), whereas BOLT-LMM, fastGWA, Regenie, and SAIGE all perform a two-step procedure, where in the first step a model is fitted to a smaller set of genome-wide markers and in the second step a larger set of imputed variants are tested for association using the model estimates from the first step (Mbatchou et al. 2021). To the best of our knowledge, although prior work has been widely successful in significantly reducing the running time of LMMs in biobank-scale data sets by using optimized implementations and heuristic approaches, there is an alarming lack of theoretical underpinnings of such methods that could provide insights on the accuracy of the heuristics that have been used to speed up LMM computations.

The aim of this work is to investigate the use of *matrix sketching* to approximately solve LMMs reducing the dimensions of the original genotype matrix while preserving the relevant properties of the original matrix for LMM computations. To that end, we propose and evaluate Matrix-Sketching LMM (MaSk-LMM).

## Results

Our work focused on both theoretical and experimental properties of matrix sketching in the context of LMMs. From a theoretical perspective, we investigated the effect of marker sketching (using the matrix $S_{2}$ of Algorithm 1) in downstream LMM computations. We do note that the theoretical properties of using the sample sketching matrix $S_{1}$ remain an important open problem for future research. From an experimental perspective, we evaluated the performance of MaSk-LMM on simulated and real-world genotypic data sets (Table 1). The experiments were performed at Purdue's Negishi and Bell clusters, consisting of Dell compute nodes with two 64-core AMD Epyc 7662 Rome processors (128 cores per node) and 256 GB of memory. The nodes run CentOS 7 and use Simple Linux Utility for Resource Management (SLURM) as the batch scheduler for resource and job management.

**Table 1.** Real data sets (coronary artery disease [CAD] and hypertension [HYP]) and simulated data sets (*D*_1_, *D*_2_, *D*_3_)

Data set	Samples	SNPs	Size (BED)
*D*_1_	10,000	265,642	634 MB
*D*_2_	100,000	265,642	6.2 GB
*D*_3_	500,000	265,642	31 GB
CAD	46,566	5,004,465	56 GB
HYP	429,480	4,599,324	453 GB

### Theoretical guarantees

A significant advantage of matrix sketching approaches is that they come with provable performance and accuracy guarantees. Indeed, this is a major objective of our work: We provide a theoretical footing to our approach by proving that at least *marker sketching* (i.e., the use of the matrix $S_{2}$ in Eqs. 2, 3) results in bounded accuracy loss with high probability. The precise statement of our result appears in Theorem 4 in Supplemental Section 2. Its proof uses a number of results from Randomized Linear Algebra along with information theoretic and probability theory inequalities.

We now present an informal statement of our results. In words, we prove that we can perform a binary hypothesis test on the parameters of an LMM by performing the computation on a marker-sketched version of the model (see Supplemental Section 1.1). This sketching procedure only increases the error probability by a small constant ϵ that can be made arbitrarily small. The sketching dimension *s*_2_ depends on ϵ, and depends linearly on *n* (the number of samples in the genotype matrix) and we also prove that this dependency is tight; that is, it *cannot be significantly reduced without catastrophically affecting the error*. We note again that this leaves as an open question the effect of sample sketching (namely, the use of the matrix $S_{1}$ in Eqs. 2, 3), which should be investigated in future work.

### Experiments: synthetic data

**Figure 1.**
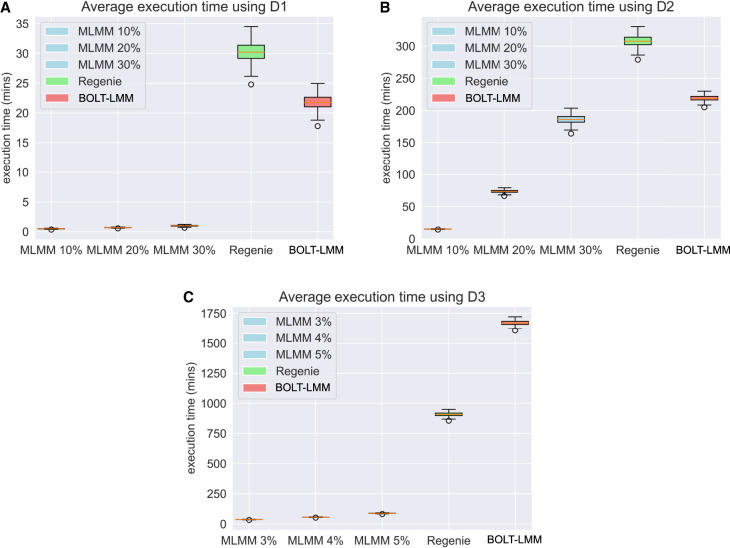
Box-and-whisker plots of the execution times across 20 identical runs of MaSk-LMM (MLMM), Regenie, and BOLT-LMM when applied to the British–Irish simulated data (265,462 SNPs) with (*A*) 10,000 samples; (*B*) 100,000 samples; and (*C*) 500,000 samples.

**Figure 2.**
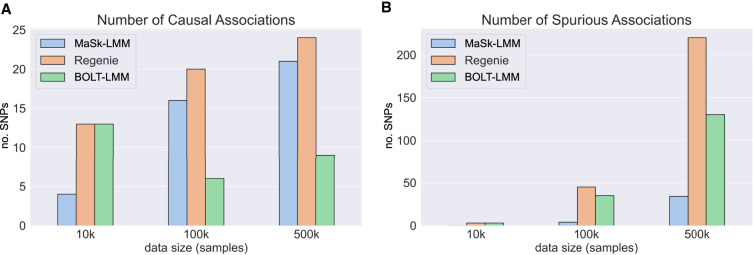
Average number of (*A*) causal and (*B*) spurious associations captured by MaSk-LMM, Regenie, and BOLT-LMM when applied to the British–Irish simulated data (265,462 SNPs and 10,000, 100,000, and 500,000 samples). *Software versions:* Regenie v3.2.5.3; BOLT-LMM v2.3.

**Figure 3.**
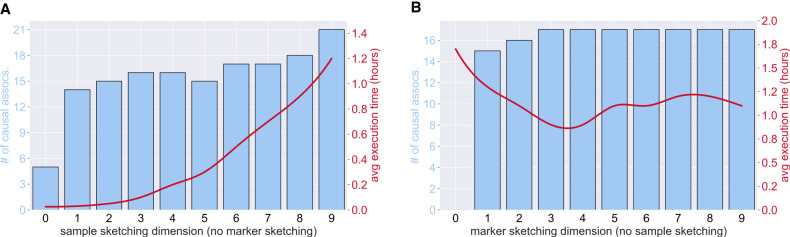
Average number of causal associations and execution time of MaSk-LMM applied to *D*_1_ (British–Irish data with 10k samples and 265k SNPs) for varied sketch dimensions across 20 identical runs. (*A*) Applying no marker sketching and varying the sample sketching from 0.1 to 1.0. (*B*) Applying no sample sketching and varying the marker sketching from 0.1 to 1.0.

For our experiments, we aimed to assess how MaSk-LMM performed in terms of execution times and accuracy of capturing causal associations (Figs. 1–3; Table 2) when compared with other methods. These evaluations are key because matrix sketching at its core is an approximation and we need to practically evaluate its shortcomings. As shown in Table 2, we measured the average execution time of MaSk-LMM, BOLT-LMM, Regenie, and FaST-LMM when applied on our simulated data sets *D*_1_, *D*_2_, and *D*_3_. We used 10% as the sketch dimension for the samples (5% for *D*_3_) and 50% as the sketch dimension for the markers when calculating the GRM. As for the reasoning behind choosing these parameters, we selected them as to not be too aggressive using very small sketch dimensions (i.e., 1%) resulting in an inaccurate sketch, but also not using too high a sketch dimension (i.e., 80%) where we may just be introducing noise and not taking full advantage of the power of matrix sketching. We can see this trade-off between accuracy and running time in Figure 3 (details in Supplemental Tables 3, 4). This choice may not be optimal for all data sets and should be tuned in accordance with the number of samples and markers. For example, for data set *D*_3_, we decided to use a 5% sample sketching, because this conservative choice allows us to have enough samples for an accurate sketch. We discuss best practices in more detail in Supplemental Section 3.

**Table 2.** Execution time (in minutes) of MaSk-LMM, Regenie, BOLT-LMM, and FaST-LMM when applied to the simulated data sets

Data set	MaSk-LMM	Regenie	BOLT-LMM	FaST-LMM
*D*_1_	1.0	30.53 (30.53)	22.00 (22)	11.00 (11)
*D*_2_	15.15	309.32 (20.42)	219.63 (14.50)	n/a^a^ (∞)
*D*_3_	88.45	911.37 (10.30)	1674.53 (18.93)	n/a^a,b^ (∞)
CAD	91.5	137.5 (1.5)	n/a^c^ (∞)	n/a^a,b^ (∞)
HYP	1063.2	1796.7 (1.69)	n/a^b^ (∞)	n/a^a,b^ (∞)

Speedup, in parentheses, achieved by MaSk-LMM compared to the other methods.

^a^Indicates no convergence after 50 h.

^b^Indicates inability to allocate space for computation.

^c^Indicates program-specific errors raised.

Results are averages over 20 identical runs. MaSk-LMM achieved speedups in execution time of 22×, 15×, and 19× over BOLT-LMM, when run on *D*_1_, *D*_2_, and *D*_3_, respectively (Fig. 1; Table 2). It also achieved speedups in execution time of 31×, 21×, and 11× over Regenie, when run on *D*_1_, *D*_2_, and *D*_3_, respectively (Fig. 1; Table 2). It also achieved a 24× speedup over FaST-LMM when run on *D*_1_ (Table 2). FaST-LMM was unable to run on the other data sets in our computing environment. MaSk-LMM utilizes Newton's method to estimate the parameters of the LMM and the number of iterations needed to converge can significantly impact the runtime, which is also dependent on the initial guess (set to 1.0 in our experiments). A better initial guess could result in faster execution times and potentially more accurate solutions.

We measured the average number of causal and spurious associations captured by MaSk-LMM, BOLT-LMM, and Regenie, when applied on the simulated data sets *D*_1_, *D*_2_, and *D*_3_ (Fig. 2; Supplemental Tables 1, 2). We report causal associations for each method using *P* < 10^−12^ to account for genome-wide significance. For each synthetic data set, we simulated 25 markers as causal with a heritability ratio of 0.5, following Yang et al. (2011). When applied to *D*_1_, MaSk-LMM performs comparably to the other two methods while being 30 × faster (Table 2). However, as we increase the sketch dimension, we do see improved performance as well as increasing running times (Supplemental Table 1). When applied to *D*_2_ and *D*_3_, MaSk-LMM outperforms BOLT-LMM, but is still slightly outperformed by Regenie, which captures slightly more causal associations. However, MaSk-LMM captures fewer spurious associations in all scenarios compared to the other methods and remains a lot faster than Regenie and BOLT-LMM (Fig. 1; Supplemental Tables 1, 2). We can see that our method steadily improves with respect to the number of causal associations that are captured as the data size grows, which illustrates the well-known fact that the performance and accuracy of matrix sketching improve when applied to larger data sets, especially when using smaller sketch dimensions (Woodruff 2014).

### Real data

We applied MaSk-LMM on data sets from complex disorders, including hypertension (HYP) and coronary artery disease (CAD) data sets. Quality control was performed in both data sets (Supplemental Section 4). In both cases, MaSk-LMM identified biologically relevant associations efficiently.

### Hypertension

We applied MaSk-LMM using a 10% sketch dimension for the samples and 50% sketch dimension for the markers on 429,480 individuals and 4,599,324 genotypes. We further reduced the computational load by generating the sketched input and GRM using the HYP data set *after* pruning. MaSk-LMM identified 812 SNPs with a *P*-value threshold of 5 × 10^−8^, accounting for genome-wide significance. We compared the significant associations with that of summary statistics from Regenie for the same data as well as an independent summary statistics of blood pressure from 342,125 individuals (Ehret et al. 2016). We assessed the qualitative significance of the associations by mapping the identified SNPs to diseases and disorders within the GWAS Catalog (Supplemental Fig. 7; Sollis et al. 2023).

Several associations are directly linked to *hypertension* and many of them are connected to *systolic* and *diastolic blood pressure*. Elevated blood pressure represents a significant and controllable contributing factor to the development and progression of various clinical manifestations associated with CAD. The impact of high blood pressure extends across the spectrum of CAD-related conditions, making it a pivotal aspect in their pathogenesis, prevention, and management. Additionally, thresholds between systolic and diastolic blood pressure are used to determine if a patient is hypertensive and their connection to cardiovascular outcomes remains a topic of interest (Flint et al. 2019). Other associations that MaSk-LMM discovered have well-established connections to HYP such as *HDL cholesterol* and *alcohol consumption* (Husain et al. 2014; Trimarco et al. 2022).

**Figure 4.**
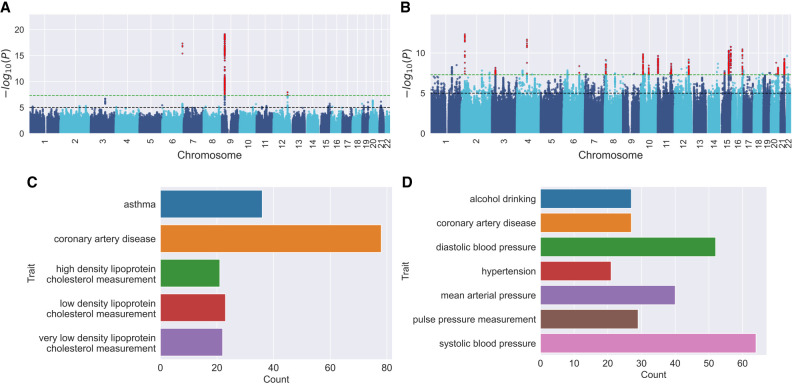
Comparing significant associations between MaSk-LMM and Regenie with Manhattan plots and mapped traits. (*A*) Manhattan plot for CAD; (*C*) Bar chart of CAD traits mapped to significant associations shared with Regenie; (*B*) Manhattan plot for HYP; (*D*) Bar chart of HYP traits mapped to significant associations shared with Regenie. The significant variants discovered by both MaSk-LMM and Regenie (11) colored in red.

We compared the performance of Regenie and BOLT-LMM with MaSk-LMM when applied to the same data set (Table 2; Fig. 4; Supplemental Figs. 5B, 8). BOLT-LMM was not able to allocate enough memory, whereas Regenie discovered 23,501 SNPs using the *P*-value threshold of 5 × 10^−8^. MaSk-LMM and Regenie had an overlap of 680 top associations (Fig. 4; Supplemental Table 5). Regenie had a similar enrichment profile to MaSk-LMM finding strong connections with systolic blood pressure, diastolic blood pressure, and HYP. In fact, we found that MaSk-LMM was able to find the same top 271 associations as found by Regenie (Supplemental Fig. 4B, up to *P* < 3 × 10^−11^), spanning multiple loci. To further investigate the power and accuracy of MaSk-LMM, we compared our findings to existing studies in the GWAS Catalog for HYP (Ehret et al. 2016) and found relevant overlap in the traits mapped to the significant associations (Supplemental Fig. 6).

### Coronary artery disease

We applied MaSk-LMM using a 30% sketch dimension for the samples and 50% sketch dimension for the markers on 46,566 individuals and 5,004,465 genotypes. We further reduced the computational time by generating the sketched input and the GRM using the CAD data set *after* pruning. MaSk-LMM identified 156 SNPs with a *P*-value threshold of 5 × 10^−8^ to account for genome-wide significance. We analyzed and assessed the significance of the associations by mapping the discovered SNPs to diseases and disorders within the GWAS Catalog (Supplemental Fig. 7; Sollis et al. 2023).

MaSk-LMM discovered many associations directly connected with *coronary artery disease*, whereas other associations are strongly linked to cardiovascular metrics such as *cholesterol measurements* and *blood pressure*. High-density lipoprotein (HDL) and low-density lipoprotein (LDL) are known to be associated with CAD (Wilson 1990), where high levels of HDL and LDL decrease and increase the risk of CAD, respectively. Similar to HYP, CAD and related cardiovascular outcomes are heavily influenced by the relationship between systolic and diastolic blood pressure (Flint et al. 2019).

We compared the performance of Regenie and BOLT-LMM with MaSk-LMM when applied to the same data set (Table 2; Supplemental Figs. 5A, 8). BOLT-LMM was not able to allocate enough memory, whereas Regenie discovered 1149 SNPs using a *P*-value threshold equal to 5 × 10^−8^. MaSk-LMM and Regenie captured the same top associations (Fig. 4; Supplemental Table 5). Regenie had a similar enrichment profile to MaSk-LMM finding strong connections with CAD, HDL, and LDL cholesterol measurements. We found the top 63 associations found by MaSk-LMM to be similar to Regenie with *P*-values up to *P* < 4 × 10^−10^ (Supplemental Fig. 4A). We also compared Regenie and MaSk-LMM increasing the sample sketching dimension to 50% and saw more significant associations with a 91% overlap with Regenie's associations. Again, we compared our findings to existing studies in the GWAS Catalog for CAD (Ehret et al. 2016) and found relevant overlap in the traits mapped to the significant associations (Supplemental Fig. 6).

## Discussion

We have developed a fast and efficient framework for linear mixed-model associations using matrix sketching. The resulting approach, MaSk-LMM, applies both sample and marker sketching to reduce the dimensions of the genotype matrix before performing LMM analysis. Such sketching speeds up the GRM computation as well as the estimation of the LMM parameters without a significant loss in accuracy. We presented theoretical support to our sketching approach by proving (Theorem 4 in Supplemental Section 2) that sketching the genetic markers (columns) of the genotype matrix results in bounded accuracy loss for the underlying LMM. To the best of our knowledge, this is the first theoretical result of its type, arguing that dimensionality reduction on the genetic marker space (which is typically massive in modern genetic data sets) is feasible without a significant loss in accuracy. We also illustrated, using synthetic data, that our method runs faster than other state-of-the-art methods while capturing almost all of the causal associations compared to the state-of-the-art methods: Few, if any, spurious associations are returned by MaSk-LMM. It is crucial to note that MaSk-LMM is a Python-based library, whereas Regenie and BOLT-LMM are both written in C++. Studies have shown that C/C++ yields a better throughput with respect to memory usage and execution time (Fourment and Gillings 2008). For completeness, we compared MaSk-LMM with FaST-LMM (Lippert et al. 2011), a Python-based tool implementing mixed models in association studies. MaSk-LMM significantly outperforms it in regards to execution time while still capturing significant associations (Table 2). The performance of MaSk-LMM can be further optimized by using pruned genotype data to accelerate the GRM computation as done in Regenie and BOLT-LMM. We have further shown that MaSk-LMM can discover biologically relevant associations when applied to data sets from complex disorders like HYP and CAD. MaSk-LMM was able to obtain the exact top associations across multiple loci, as the state-of-the-art method Regenie with *P*-values up to 4 × 10^−10^, while taking a fraction of Regenie's computation time (Table 2; Supplemental Fig. 4).

MaSk-LMM is an important advance and contribution to the space of genomics, specifically when conducting GWAS. Biobank-scale data sets spanning hundreds of thousands of individuals offer unprecedented opportunities to discover novel genetic loci associated with complex human traits and disease risk. However, they also present a computational challenge and burden. Using matrix sketching, we are able to harness the quality and richness of biobank-scale data, while also alleviating the computational burden by reducing their dimensionality.

Matrix sketching is a well-explored technique with robust theoretical underpinnings in Theoretical Computer Science and Applied Mathematics. However, its adoption in healthcare and life science applications remains limited. The primary reason for this limited acceptance is that the prevailing approach in these fields emphasizes accumulating ever-increasing volumes of data, whereas matrix sketching appears to reduce data sizes, at least at first glance. In this work, we demonstrate that matrix sketching can be a powerful and meaningful tool, showcasing the potential and significance of matrix sketching in healthcare and life science applications. By embracing matrix sketching, we have managed to achieve significant benefits that mitigate concerns about data reduction.

Even though MaSk-LMM illustrates the power of approximate computations using matrix sketching in the context of LMMs, it is not without its limitations. First of all, there is a trade-off between the sketching dimension, the number of causal associations captured, and its running time (Supplemental Section 3; Supplemental Tables 3, 4; Figs. 2, 3). Using more aggressive sketching and reducing the number of retained markers or samples (parameters *s*_1_ and *s*_2_ in Algorithm 1) to 5%–10% of the original values *m* and *n*, reduces the running time. However, it also worsens the quality of the approximation, resulting in fewer causal associations captured and potentially more spurious associations. This issue becomes less prevalent as the data set size increases, because the abundance of markers and samples helps improve the quality of sketching even when using smaller sketch dimensions. Additionally, our current implementation has not incorporated the leave-one-chromosome-out cross-validation (LOCO) to correct for proximal contamination, a phenomenon that could result in loss of power if the candidate marker is included in the GRM (Yang et al. 2014). However, in our setting, the input is sketched and the GRM computation operates on a much smaller matrix, which seems to mitigate this issue, at least in our empirical evaluations. Other future research directions that could improve our framework include taking advantage of sparsity in our computations, improving data management, as well as implementing our methods in an environment that is more suitable for high-performance computing with biobank-scale data, like C++ with Intel's OpenMPI supporting libraries.

## Methods

### Mixed-model association

LMMs are formed using the following simple linear model:

(1) y=Xβ+Zu+e,

where $y\in R^{n}$ is the measured phenotype (response); $X\in R^{n\times k}$ is the matrix of the *k* covariates (e.g., principal components, age, sex, etc.) with the corresponding vector of fixed effects ***β*** ∈ ℝ^*k*^; $Z\in R^{n\times m}$ is the genotype matrix of *n* individuals genotyped on *m* genetic markers with $u\in R^{m}$ being the corresponding genetic effects vector; and $e\in R^{n}$ is the error vector or the component of y which cannot be explained by the model. We use bold letters for vectors and matrices; a vector $x\in R^{n}$ is an *n*-dimensional real vector, whereas a matrix $X\in R^{n\times m}$ is an *n* × *m* real matrix. We assume u and e are independent vectors and moreover that $u∼N(0,\sigma_{g}^{2}\;I_{m})$ and $e∼N(0,\sigma_{e}^{2}\;I_{n})$ with scalars $\sigma_{g}^{2}$ and $\sigma_{e}^{2}$ being the heritable and nonheritable components of u and e, respectively. We use the notation N(μ,Σ) to denote a multivariate normal distribution with mean vector μ and covariance matrix Σ. $I_{n}$ denotes the *n* × *n* identity matrix. In the LMM setting, some form of maximum likelihood estimation is used to estimate the random and fixed effects of the model in order to identify genetic associations while correcting for confounding effects.

### MaSk-LMM

Our approach, MaSk-LMM, mitigates the computational complexity of LMMs by using sample and marker sketching on the input genotype matrix Z, as well as on the response vector y. This allows us to significantly reduce the dimensions of the genotype matrix, as well as of the relatedness or kinship matrix (GRM). As discussed in the introduction, sketching reduces the dimensions of the input while maintaining sufficient information to approximate the functions of the original input accurately. Let $S_{1}\in R^{s_{1}\times n}$ and $S_{2}\in R^{m\times s_{2}}$ be two sketching matrices, with *s*_1_ ≪ *n* and *s*_2_ ≪ *m*. Here *s*_1_ and *s*_2_ are the sketching dimensions and are user-controlled parameters. Simple constructions for $S_{1}$ and $S_{2}$ are to have their entries drawn in independent identical trials from a Gaussian distribution of zero mean and variance 1/*s*_1_ and 1/*s*_2_, respectively. We can then use $S_{1}$ and $S_{2}$ to sketch the input genotype matrix as follows:

(2) $$Z_{s_{1},s_{2}}=S_{1}ZS_{2}\in R^{s_{1}\times s_{2}}.$$

$Z_{s_{1},s_{2}}$ is computed in blocks so the entire original input does not need to be loaded into memory, alleviating a portion of the computational burden of this approach. Notice that $Z_{s_{1},s_{2}}$ is now a much smaller *s*_1_ × *s*_2_ matrix which can be used in downstream computations instead of Z. For example, we can approximate the GRM as follows:

(3) $$K=Z_{s_{1},s_{2}}Z_{s_{1},s_{2}}^{⊤}=S_{1}ZS_{2}S_{2}^{⊤}Z^{⊤}S_{1}^{⊤}\in R^{s_{1}\times s_{1}}.$$

**Figure 5.**
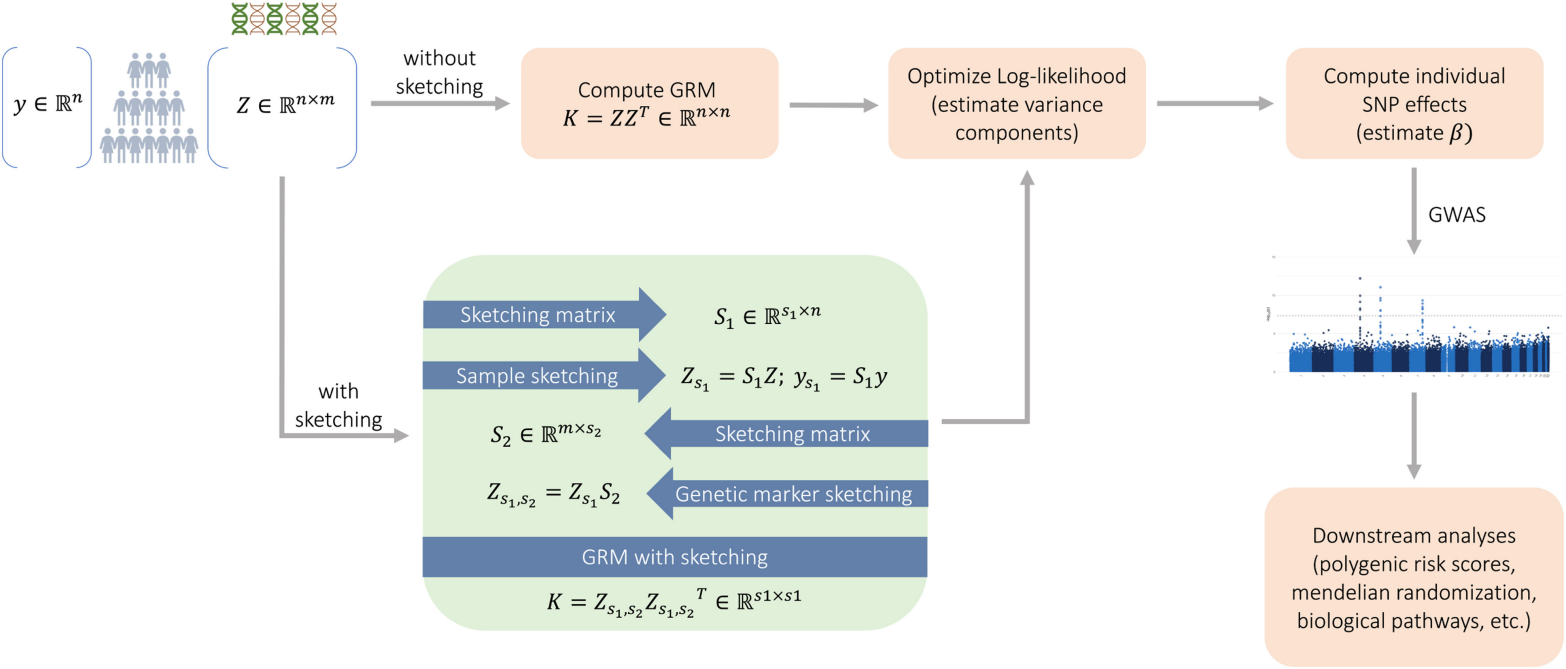
The MaSk-LMM framework. We use sketching to speed up the standard pipeline of LMM computations (peach). Our alternative pipeline uses sketching on both the sample and marker space of the genotype matrix Z (see Eqs. 2, 3) to speed up computations (green).

We also sketch the *n*-dimensional response vector y to construct the *s*_1_-dimensional response vector $y_{s_{1}}=S_{1}y$ to be used in downstream computations instead of y. It is worth noting that there is a long line of research on matrix sketching methods, including Gaussian sketching, the use of the subsampled randomized Hadamard transforms, the count-min sketch, etc., and its application in human genetics (Bose et al. 2019, 2021, 2023). In our work, we evaluated both the count-min sketch and the Gaussian sketch. Both methods performed similarly and we chose to report results on Gaussian sketching only, because it is conceptually simpler as well as easier to implement and theoretically analyze. See Woodruff (2014) for a discussion of other sketching methods and their theoretical properties. Figure 5 summarizes our framework and Algorithm 1 provides a high-level overview of our approach.

Algorithm 1: **MaSk-LMM**

1: **Input:** Normalized genotype matrix $Z\in R^{n\times m}$, normalized response vector $y\in R^{n}$, sample sketch dimension *s*_1_, marker sketch dimension *s*_2_

2: **Output:** Estimated variance components ($\tau ,\sigma_{g}^{2}$) and test statistics

3: $Z_{s_{1}}=S_{1}Z\in R^{s_{1}\times m}$, where $S_{1}(i,j)∼N(0,s_{1}^{-1})$ for *i* = 1…*s*_1_, *j* = 1…*n*

4: $y_{s_{1}}=S_{1}y\in R^{s_{1}}$ ($S_{1}$ as above)

5: Compute the top principal components (PCs) of $Z_{s_{1}}$ to use as covariates; add any other covariates; return $X\in R^{s_{1}\times k}$ as the covariate matrix

6: $K=S_{1}ZS_{2}S_{2}^{⊤}Z^{⊤}S_{1}^{⊤}\in R^{s_{1}\times s_{1}}$, where $S_{2}(i,j)∼N(0,s_{2}^{-1})$ for *i* = 1…*m*, *j* = 1…*s*_2_

7: Estimate variance components ($\tau ,\sigma_{g}^{2}$) using Newton's method on the log-likelihood function (see Supplemental Section 1.1 and Supplemental Algorithm 1 for details)

8: Set $V=\sigma_{g}^{2}\;H_{\tau }$, with $H_{\tau }=\frac{1}{m}K+\tau I_{n}$

9: *For each* column $Z_{test}$ in $Z_{s_{1}}$:

10: $\chi^{2}=\frac{{\left(Z_{test}V^{-1}y_{s_{1}}\right)}^{2}}{Z_{test}^{⊤}V^{-1}Z_{test}}$

11: **end**

### Data

Our experimental proof-of-principle evaluation seeks to demonstrate that sketching is a viable approach for LMMs. We chose to evaluate our algorithm on real and simulated data in order to show both run time and accuracy guarantees of MaSk-LMM when compared to the current state-of-the-art. We used genomic and clinical records from UK Biobank (UKB) as per application 95318 for our analyses.

#### Simulated genotypes

The synthetic data were generated from two ancestral backgrounds, Irish and British, using a “mosaic-chromosome” scheme modified from Loh et al. (2015). The general concept is to take a small set of individuals that are genetically distinct and generate artificial individuals by sampling their genomes. We began by selecting all individuals with British and Irish ancestries from the UKB data after performing quality control and pruning, thus resulting in a data set of 435,655 individuals and 265,642 SNPs. We then filtered the samples based on their ancestries inferred from SNP data (using the top two PCs, see Supplemental Fig. 1A) to ensure that the two groups were genetically distinct. We selected 100 samples from that subset of individuals to treat as the founders or ancestors from which to generate the artificial individuals. We divided the genome into consecutive segments of 2000 variants and generated *unrelated* individuals by selecting each segment from one of the 100 ancestors chosen at random and simulated *related* individuals by selecting the segments from a smaller number of ancestors according to the degree of relatedness. This process is done for both the Irish and British populations (see Supplemental Fig. 1B). Finally, we used GCTA tools (Yang et al. 2011) to simulate quantitative and binary traits for our simulated individuals.

#### Real genotypes

The real genotypes were extracted from the UKB (as per approved research ID 95318) for HYP and CAD. After performing quality control using PLINK v2 (Chang et al. 2015), the HYP data set had 429,480 samples and 4,599,324 high-quality SNPs (see Supplemental Sections 4 and 5 for details). The CAD data set had 46,566 samples and 5,004,465 SNPs. MaSk-LMM's GRM was computed after pruning these data sets after quality control, with *r*^2^ > 0.8. The UKB data sets were created after curating ICD-10, ICD-9, and self-reported codes to more meaningful phenotypes (see Supplemental Section 5 for details). We computed the top 20 principal components using TeraPCA (Bose et al. 2019).

### Software availability

A Python implementation of MaSK-LMM is available at GitHub (https://github.com/IBM/mask-lmm) and as Supplemental Code.

## Supplementary Material

Supplement 1

Supplement 2
