# Supplementary figures and images for: Matrix sketching framework for linear mixed models in association studies
Source: Genome Res. 2024 Sep;34(9):1304–11. doi: 10.1101/gr.279230.124 (PMC11529869; doi:10.1101/gr.279230.124)

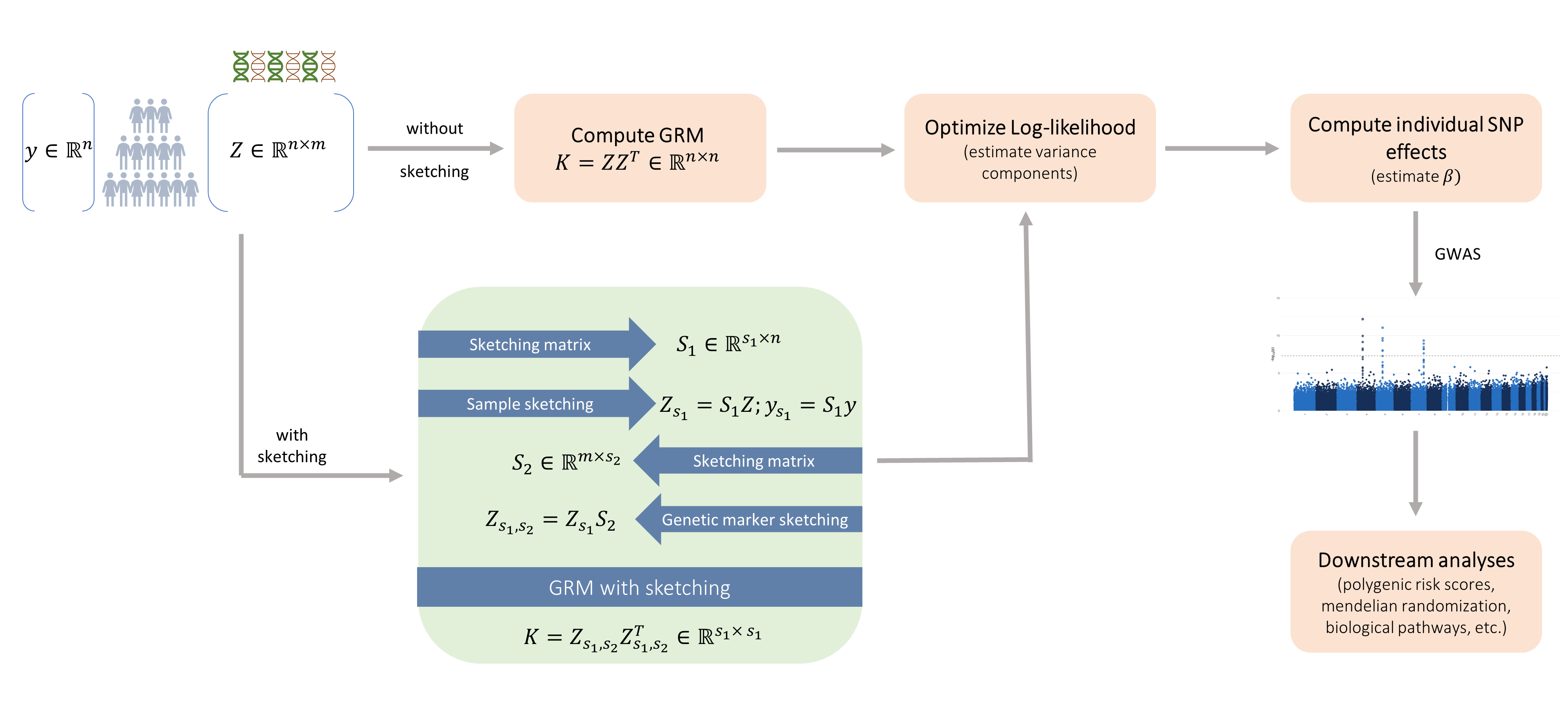

Supplement: Supplement 1 [file Supplemental_Code.zip › mask-lmm-main/images/masklmm_overview.png]
